# Efficacy of Laryngeal Tube versus Bag Mask Ventilation by Inexperienced Providers

**DOI:** 10.5811/westjem.2020.3.45844

**Published:** 2020-04-16

**Authors:** Danielle Hart, Brian Driver, Gautham Kartha, Robert Reardon, James Miner

**Affiliations:** Hennepin Healthcare, Department of Emergency Medicine, Minneapolis, Minnesota

## Abstract

**Introduction:**

Bag mask ventilation (BMV) and extraglottic devices (EGDs) are two common methods of providing rescue ventilation. BMV can be difficult to perform effectively, especially for inexperienced providers and in patients with difficult airway characteristics. There is some evidence that the laryngeal tube (LT) can be successfully placed by inexperienced providers to provide effective ventilation. However, it is unclear whether ventilation provided by LT is superior to that of BMV, especially in the hands of inexperienced airway providers. Therefore, we aimed to compare ventilation efficacy of inexperienced airway providers with BMV versus LT by primarily measuring tidal volumes and secondarily measuring peak pressures on a simulated model.

**Methods:**

We performed a crossover study first year emergency medicine residents and third and fourth year medical students. After a brief instructional video followed by hands on practice, participants performed both techniques in random order on a simulated model for two minutes each. Returned tidal volumes and peak pressures were measured.

**Results:**

Twenty participants were enrolled and 1200 breaths were measured, 600 per technique. The median ventilation volumes were 194 milliliters (mL) for BMV, and 387 mL for the laryngeal tube, with a median absolute difference of 170 mL (95% confidence interval [CI] 157–182 mL) (mean difference 148 mL [95% CI, 138–158 mL], p<0.001). The median ventilation peak pressures were 23 centimeters of water (cm H_2_O) for BMV, and 30 cm H_2_O for the laryngeal tube, with a median absolute difference of 7 cm H_2_O (95% CI, 6–8 cm H2O) (mean difference 8 cm H_2_O [95% CI, 7–9 cm H_2_O], p<0.001).

**Conclusion:**

Inexperienced airway providers were able to provide higher ventilation volumes and peak pressures with the LT when compared to BMV in a manikin model. Inexperienced providers should consider using an LT when providing rescue ventilations in obtunded or hypoventilating patients without intact airway reflexes. Further study is required to understand whether these findings are generalizable to live patients.

## INTRODUCTION

Rescue ventilation, performed for apneic or hypoventilating patients and after failed intubation attempts, is an important skill for emergency airway management. Bag mask ventilation (BMV) and the use of extraglottic devices (EGDs) are two common methods of providing rescue ventilation.

BMV, long the gold standard, can be difficult to perform effectively and requires proper technique to ensure sufficient ventilation. 1–5 In a study of first-year anesthesia residents, only 17% were able to provide effective BMV on anesthetized patients following a traditional 36-hour BMV and endotracheal intubation (ETI) course. 2 BMV can prove to be especially difficult in patients with older age, obesity, lack of teeth, a beard, a higher Mallampati class, or history of snoring. 6,7

EGDs provide similar ventilation to endotracheal tubes, 8–10 are easy to place, and are often used for emergency ventilation. The laryngeal tube (LT), a type of EGD, can be successfully placed by inexperienced providers to provide effective ventilation. 3,11–13 However, there is conflicting evidence on whether ventilation provided by LT is superior to that of BMV, and it is unknown whether an efficacy difference exists in the hands of inexperienced airway providers or those who infrequently perform emergency ventilation, a group that requires an easy and effective method to maintain oxygenation. Proper BMV may require skill acquisition and maintenance that would be difficult for providers that rarely perform the procedure; however, training and skill acquisition for both BMV and EGD placement is important. Studies examining minute ventilation as well as those using a subjective outcome of “adequate ventilation” as judged by the care provider have yielded conflicting results when examining the efficacy of LT versus BMV. 3,8,14,15

We therefore aimed to compare ventilation efficacy of inexperienced airway providers with BMV versus LT on a simulated model. Our primary outcome was measured tidal volume, and our secondary outcome was peak pressure. We hypothesized that LT would produce higher tidal volumes and peak pressures than BMV.

## METHODS

We performed a crossover study, including first year emergency medicine (EM) residents and third and fourth year medical students. Twenty participants were enrolled, all inexperienced in airway management: 12 medical students, seven first year EM residents, and one paramedic student. We chose these participants because they were largely inexperienced in basic airway management. The local institutional review board declared this study exempt from review; all participation was voluntary.

To teach the basics of BMV and LT insertion, participants listened to a brief introductory lecture discussing basic airway management and watched a standardized, four-minute video that described best practices for BMV and LT insertion and use. The two-handed thenar eminence (TE) BMV technique was taught due the superiority of this technique when compared to the one-handed or two-handed E-C technique; 16,17 in this technique, the thenar eminences rest on the mask, and the fingers lift the ramus of the mandible upward into the mask to create a seal (Appendix). For LT insertion, participants were instructed to perform a jaw lift, insert the LT deeply, then withdraw the tube slowly during ventilation, until adequate ventilation was achieved.

After a period of unstructured hands-on practice (which included the same length of time, manikins, airway equipment, and instructor availability for all participants), participants performed both techniques in random order. They were given a standard adult size facemask and a #4 King LT. We used a manikin to compare the effectiveness of each technique (TruCorp AirSim Combo X; Belfast, Ireland); the esophagus and cricothyroid membrane apertures were taped closed; a 3 liter reservoir bag (Intersurgical; East Syracuse, NY) was used to simulate inflation and deflation of a lung. The manikin was inspected for any tears or disruptions prior to data collection to ensure there were no detectable areas that would result in an air leak. A mechanical ventilator, connected with standard ventilator tubing to the facemask or laryngeal tube, (Viasys LTV 1200; Vyaire Medical, Mettawa, IL), delivered a tidal volume of 500 milliliters (mL) at 15 breaths per minute, and measured peak pressure and returned tidal volumes. We used a ventilator rather than manual bagging in order to standardize the volume delivered, allowing comparisons between devices

After LT insertion or establishing a facemask seal, five breaths were administered to inflate the reservoir bag; then, the participants performed each technique for two minutes. The LT or mask position could be adjusted at any time to maintain the best possible ventilation. Participants were able to see the reservoir bag inflating and deflating during the ventilation; no other real-time feedback or assistance was provided. We recorded the tidal volume and peak pressure for each ventilation. This essentially compared the ability of subjects to achieve an airway seal with a two-hand thenar eminence technique on a mask compared to placement of the LT.

The primary outcome was returned tidal volume; the secondary outcome was peak pressure. These parameters are indicative of the effectiveness of the airway technique and have been used in prior research. 17 Assuming delivered volumes of about 400 mL (with a standard deviation of approximately 75 mL), we estimated 20 subjects would be required to detect a 50 mL difference in volumes delivered by the two techniques. Using the Shapiro-Wilk normality test, we determined that neither tidal volume and peak pressure values were normally distributed. Therefore, we compared the volumes and pressures for the two techniques by calculating the median difference between groups. The mean values are also presented. We used Stata (version 15.1, College Station, TX) for all data analysis.

## RESULTS

**Figure 1:**
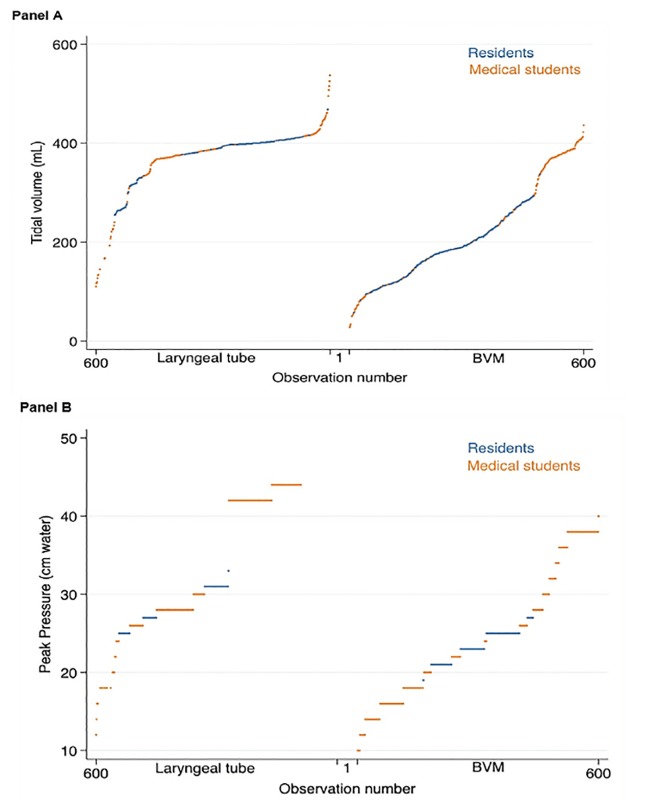
Tidal volume and peak pressure. This figure displays each tidal volume (panel A) and peak pressure (panel B) measurement for the 1,200 breaths administered, sorted in ascending order and by group. *mL*, milliliters; *cm*, centimeters; *BVM*, bag mask ventilation.

All participants performed both techniques; 1200 breaths were measured, 600 per technique. The median ventilation volumes were 194 mL for BMV, and 387 mL for the laryngeal tube, with a median absolute difference of 170 mL (95% confidence interval [CI], 157–182 mL) (mean difference 148 mL [95% CI, 138–158 mL], p<0.001). The median ventilation peak pressures were 23 centimeters of water (cm H_2_O) for BMV, and 30 cm H_2_O for the laryngeal tube, with a median absolute difference of 7 cm H_2_O (95% CI, 6–8 cm H_2_O) (mean difference 8 cm H_2_O [95% CI, 7–9 cm H_2_O], p<0.001). Volumes and pressures achieved by training level are displayed in the Table. Performance of each participant in each technique is presented in Figure 1.

## DISCUSSION

Although BMV is often the first-line method of emergency ventilation, there is growing evidence supporting the use of LTs and other EGDs in airway management, including those with out of hospital cardiac arrest (OHCA), and those requiring advanced airway management in the out-of-hospital setting, ED, or during general anesthesia. Prior literature suggests that the LT has a high rate of successful placement and adequate ventilation. 3,18–21 What is less clear is how the LT compares to BMV in the hands of inexperienced providers.

In our study, we found significantly higher ventilation volumes and peak pressures when using the LT compared to BMV for medical students and first year EM residents. This supports previous work of Kurola, who found significantly higher minute ventilation with LT compared to BMV in a simulation model with emergency medical technician (EMT) students, and of Roth, who found that the LT was subjectively more effective than BMV in OHCA patients managed by volunteer EMTs. 3,8 There are, however, a few studies that have not found differences in ventilation provided by LT versus BMV. Kurola later found that both LT and BMV were equally effective in ventilating and oxygenating anesthetized patients in the controlled setting of the operating room (OR) in the same study population as his simulation-based study. In addition, Fiala et al found no difference between BMV and the LT for ventilating OHCA patients in a multicenter randomized study of EMT-led airway management. 14,15

Considering other EGDs, our findings are also consistent with multiple prior studies of inexperienced providers using laryngeal mask airways (LMA), all of which concluded that inexperienced airway providers (including nurses, nursing students, dental students, and other volunteers with no prior experience) can provide better ventilation with the LMA than with BMV (with or without a concomitant oropharyngeal airway). 5,22–26 One study looking specifically at obese patients found that medical students were able to establish effective ventilation more quickly with the LMA than with BMV. 27 A few studies that contradict these findings used more experienced providers, highlighting the need for experience and practice in order to ventilate with BMV effectively. 2,28–30

EGDs are essential for emergency ventilation in patients who are known to have difficult mask ventilation, such as those with beards, morbid obesity, or a history of snoring. 6,7,27 In cardiac arrest patients, EGDs result in a lower incidence of gastric insufflation and regurgitation than BMV. 3,5,26,31 With prior conflicting results regarding the efficacy of LT versus BMV in providing superior ventilation, our results add support to the assertion that the LT may be a better choice than BMV in obtunded or hypoventilating patients without intact airway reflexes for inexperienced providers who have not developed effective BMV techniques. 1–5 Knowing that LMAs have also shown to result in superior ventilation to BMV in the hands of inexperienced providers, it is possible that when an inexperienced provider encounters a patient who requires emergency ventilation, an EGD may be preferred to BMV, because this device requires less practice and skill, and likely allows higher tidal volume and peak pressure, enabling better overall ventilation. Further study is required to determine whether the findings in our study of LT being superior to BMV for inexperienced providers is generalizable to live patients.

## LIMITATIONS

Our study included a convenience sample of subjects, who may have volunteered for our study due to their perceived increased or decreased skill compared to their overall cohort. There are also inherent differences between manikins and actual patients, including a taped-off esophagus. While this differs from human anatomy, closing off the esophagus was necessary to accurately measure delivered and returned tidal volumes and pressures for this study. While a human model would be preferred, unfortunately there is no practical way to compare BMV with LT insertion for inexperienced providers during actual emergency airway management. Therefore, these data should serve as a surrogate that reflects the increased difficulty in obtaining a mask seal compared to inserting an EGD in the clinical environment. Although further human study in emergency airway management may not be feasible in an emergency setting, further study of inexperienced medical students and residents could be performed in the more controlled environment of the operating room. This could then account for additional variables that may occur in live patients, such as variations in human anatomy and differences in lung compliance. Similarly, use of a ventilator rather than a resuscitation bag does not mirror real-world practice, but enabled standardization of tidal volumes between groups, so that differences in measured volume and pressure were due to differences in laryngeal tube or BMV technique rather than differences in bag squeezing. We did not measure skill retention, which could be an area of future exploration. Finally, we had a small sample size of 20 participants; although this sample size provided power to detect a 50 mL difference, a larger sample size would provide further mitigation of type 2 error.

## CONCLUSION

Inexperienced airway providers were able to provide higher ventilation volumes and peak pressures with the LT when compared to BMV in a manikin model. Inexperienced providers should consider using an LT when providing rescue ventilations in obtunded or hypoventilating patients without intact airway reflexes. Further study is required to understand whether these findings are generalizable to live patients.

## Supplementary Information



## Figures and Tables

**Table:** Median and mean volume delivered, by training level and technique.

Training level	Laryngeal tube	Bag-mask ventilation
	Volume (mL)	
	
First year resident	398 (318 to 402); 367 (54)	194 (161 to 232); 194 (53)
Medical student	382 (370 to 405); 382 (26)	227 (143 to 347); 243 (109)
	
	Pressure delivered (cm H_2_O)	
	
First year resident	29 (26 to 31); 29 (3)	23 (22 to 25); 23 (1.6)
Medical student	36 (28 to 42); 35 (8)	25 (17 to 35); 26 (9)

*mL*, milliliters; *cm H_2_O*, centimeters of water.

All values are median (interquartile range); mean (standard deviation).
