# Supplementary figures and images for: Efficacy of Laryngeal Tube versus Bag Mask Ventilation by Inexperienced Providers
Source: West J Emerg Med. 2020 Apr 16;21(3):688–93. doi: 10.5811/westjem.2020.3.45844 (PMC7234713; doi:10.5811/westjem.2020.3.45844)

**Appendix**


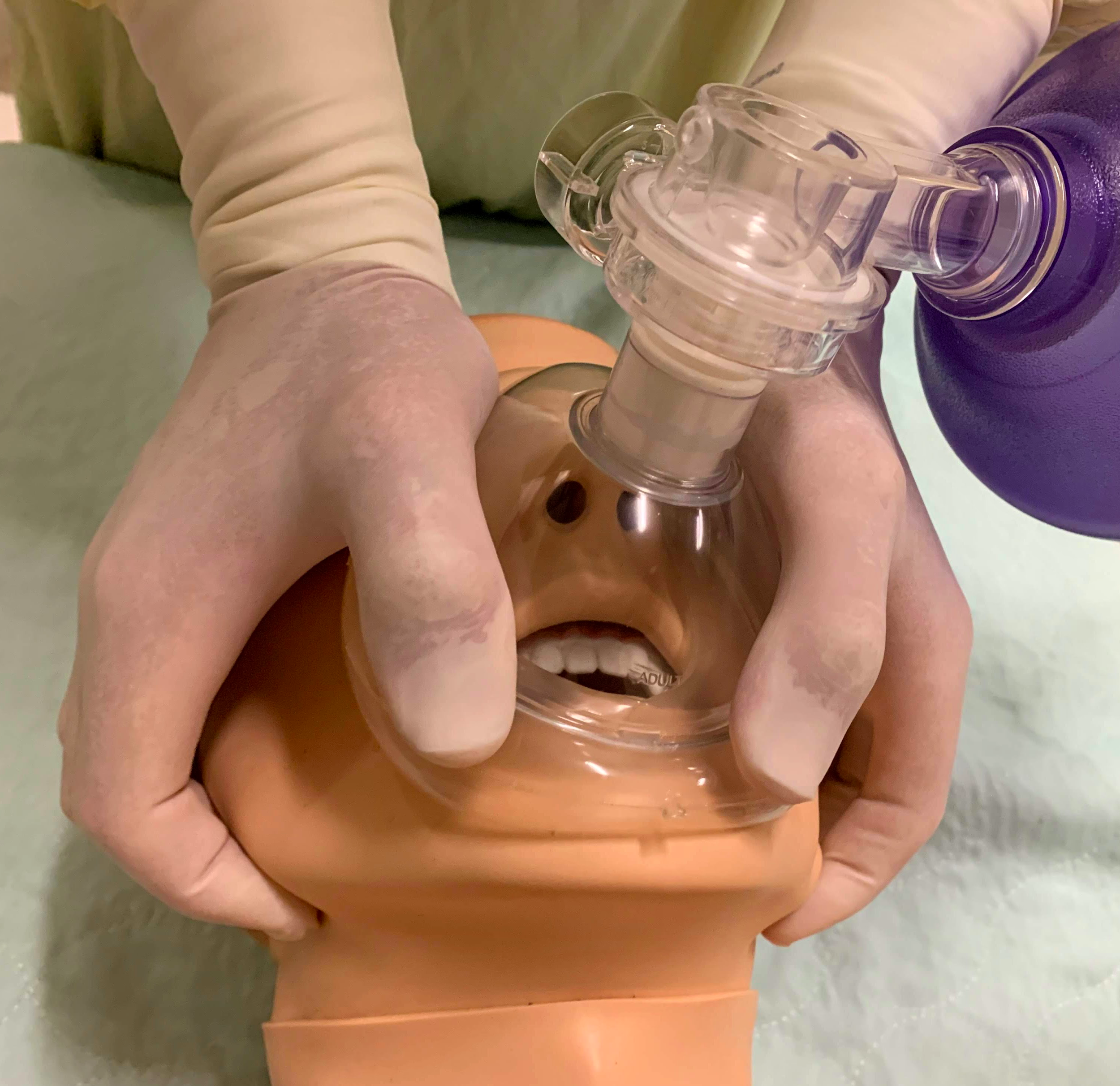


**Image.** Two-handed thenar eminence technique for bag mask ventilation.

Supplement: Supplementary file 1 [file wjem-21-688-s001.docx]
